# Population genetic structure analysis and effect of inbreeding on body weights at different ages in Iranian Mehraban sheep

**DOI:** 10.1186/s40781-014-0031-3

**Published:** 2014-12-10

**Authors:** Roya Yavarifard, Navid Ghavi Hossein-Zadeh, Abdol Ahad Shadparvar

**Affiliations:** Department of Animal Science, Faculty of Agricultural Sciences, University of Guilan, P. O. Box: 41635–1314, Rasht, Iran

**Keywords:** Fat-tailed sheep, Growth traits, Inbreeding depression, Mating program, Pedigree analysis

## Abstract

The objective of this study was to describe the population structure and inbreeding, and to quantify their effects on weights at different ages of Mehraban sheep in Iran. The analysis was based on the pedigree information of 26990 animals and 10278 body weight records from birth to yearling age. Data and pedigree information were collected during 1994 to 2011 by the breeding station of Mehraban sheep. The population structure was analyzed using the CFC program. Inbreeding of all animals was calculated by INBUPGF90 program. All animals were grouped into three classes according to their inbreeding coefficients: the first class included non-inbred animals (F=0); and the second and third classes included inbred animals (0<*F*<0.05 and F≥0.05, respectively). The average inbreeding in Mehraban sheep was 1.69%. Founder equivalent (f_e_) values were estimated to be 4244, 3116 and 2965 during 1994-1999, 2000-2005 and 2006-2011, respectively. The effective population sizes (N_e_) were 363, 5080 and 5740 during 1994-1999, 2000-2005 and 2006-2011, respectively. Generation interval was 2.15 years for this breed of sheep. Regression coefficients of birth weight, 3-month weight, 6-month weight and yearling weight on lamb inbreeding were estimated to be -6.34±0.69, -14.68±5.33, 48.00±9.43 and 98.65±15.65, respectively. Both positive and negative inbreeding effects were found in the current study. The utilization of a program for designed mating system, in the present flock, could be a suitable approach to keep the level of inbreeding under control.

## Background

During the past 50 years, the indigenous genetic reserves are in a critical stage all over the world due to large changes in production systems, change of market demand and intercourse of domestic animals with other breeds. Along with increase in genetic progress, maintaining genetic diversity in the population is very important to adapt with the economic and environmental changes in the future and ensure long-term response to selection for traits that are very important [1]. Effective population size (N_e_) is a criterion for determining similarity between alleles of the loci so that there was a common ancestor and determines the level of inbreeding and reduced rate of genetic variation due to gene random drift [2]. Intensive use of a few breeding animals, where the selection intensity is high, could result in greater rates of inbreeding in the population. Therefore, a small number of seedstock, with a strong family relationship, is responsible for the maintenance of almost the whole genetic pool in the population. This is an aspect of great influence in the genealogical analysis of a population structure, because of its effect on the probability of genes lost between generations and the consequent reduction in genetic variability [3].

Discrepancies in ancestral origins and migration events are important causative factors explaining genetic differences between current populations [4-6]. Hence, the optimal management of population is essential in order to prevent from decrease in diversity. Estimation of parameters such as effective population size, inbreeding and coancestry are depended significantly on the genealogy information. Measurement of the effect of inbreeding on economic traits is important in order to estimate the magnitude of change associated with increases in inbreeding. It is apparent that different breeds and populations, as well as different traits vary in their response to inbreeding. Some populations may show a very pronounced effect of increased inbreeding for a trait, whereas others may not display much of an effect [7,8]. The rate of inbreeding needs to be limited to maintain diversity at an acceptable level, so that genetic variation will ensure that future animals can respond to changes in the environment and to selection. Without genetic variation, animals cannot adapt to these changes [9]. Commonly, negative inbreeding effects, or inbreeding depression, are thought to most frequently occur because of an increase in frequencies of recessive alleles that adversely affect the traits of interest [10]. The increased frequency of recessive alleles leads to a larger number of individuals that are homozygous for the recessive alleles, whereas in non-inbred populations, the recessive allele would more frequently be masked by an advantageous dominant allele [11].

One of the most important breeds of Iranian sheep is Mehraban sheep which is reared in Hamedan province. This breed is adapted to harsh climate and rocky environments in the western regions of Iran. The Mehraban is a fat-tailed carpet wool sheep with light brown, cream or grey color, dark face and neck and primarily used for meat production [12]. The objective of this study was to describe the population structure and inbreeding, and to quantify their effects on weights at different ages of Mehraban sheep in Iran from 1994 to 2011.

## Methods

### Experimental design and animals

Data set and pedigree information used in this research were collected from the breeding station of Mehraban sheep (Hamedan, Iran) during 1994–2011. The traits included were: Birth weight (BW, n= 10287), 3-month weight (WW, n= 6735), 6-month weight (6 MW, n= 4778), 9-month weight (9 MW, n= 3139) and yearling weight (YW, n= 1985). Ewes were randomly exposed to the rams at about 18 months of age. Matings were controlled and single-sire pens were used allocating 10–15 ewes per ram. Ewes were kept in the flock up to 7 years of age. Ewes usually give births to lambs three times every two years. All lambs were weighed and ear tagged within 12 hours after the birth. Lambs are weaned at approximately 90 days of age. Flocks were grazed during the daytime and housed at night. The lambs were kept indoors and fed manually during the winter. Summary of pedigree information used in this research is presented in Table 1. Also, descriptive statistics of data used in the analysis are shown in Table 2.

**Table 1:** **Summary of pedigree information for Mehraban sheep**

**Item**	**N**	**%**
Individuals in total	26990	100%
Sires	405	15%
Dams	8114	30%
Individuals with known sire	304	11%
Individuals with known dam	11339	42%
Individuals with known sire and dam	3683	14%
Individuals with progeny	18472	68%
Inbred individuals	18872	70%

**Table 2:** **Characteristics of data set for Mehraban sheep**

	**BW**	**WW**	**6 MW**	**9 MW**	**YW**
Number of records	10278	6735	4778	3139	1985
Mean (kg)	3.69	22.16	36.13	45.48	52.70
Standard deviation (kg)	0.76	4.26	6.09	6.43	6.74
Coefficient of variation (%)	20.60	19.22	16.85	14.14	12.79
Minimum (kg)	1.35	8.92	15.06	25.85	28.46
Maximum (kg)	6.03	35.74	58.50	64.91	75.48

*BW*: birth weight, *WW*: 3-month weight, *6 MW*: 6-month weight, *9 MW*: 9-month weight, *YW*: yearling weight.

### Statistical and genetic analyses

The CFC program [13] was used to calculate pedigree statistics and genetic structure analysis of the population. To characterize the population structure, variation changes in inbreeding (ΔF), average coancestry (AC), effective population size (N_e_), generation interval (L) and parameters derived from the method of analysis of gene origin probability were calculated. The parameters related to the method of analysis of gene origin probability were: founder equivalent (f_e_), founder genome equivalent (f_g_), effective number of non-founder (N_enf_), average number of discrete generation equivalents (G_e_), maximum number of discrete generation equivalents (MaxG_e_) and minimum number of discrete generation equivalents (MinG_e_). Animals were grouped based on their birth years into three classes (1994–1999, 2000–2005, and 2006–2011). This classification was necessary for the CFC program to compute the genetic structure parameters of the population.

To account for unequal founder representation, Lacy [14] estimated the effective number of founders (f_e_) as:

$$ {f}_e={\left[{\displaystyle \sum_{i=1}^f{p}_i^2}\right]}^{-1} $$

Where **p**_**i**_ is the expected proportional genetic contribution of founder **i**, calculated by the average relationship of the founder to each animal in the current population, and **f** is the total number of founders. The parameter f_e_ indicates the number of equally contributing founders that would produce the same level of genetic diversity as that observed in the current population [15].

Bottlenecks, genetic drift and unequal founder contributions which have a greater impact in small populations can be quantified using the founder genome equivalent (f_g_) as follows:

$$ {f}_g={\left[{\displaystyle \sum_{i=1}^f\frac{{\displaystyle {p}_i^2}}{r_i}}\right]}^{-1} $$

Where **r**_**i**_ is the expected proportion of founder i’s alleles that remain in the current population and can take on a value of 0.5 if one allele is present or 1.0 if two alleles are present, **p**_**i**_ is the expected proportional genetic contribution of founder **i**, and **f** is the number of contributing founders [14].

Average number of discrete generation equivalents was determined for total pedigree of each flock according to the following equation:

$$ {G}_e=\frac{1}{N}{\displaystyle \sum_{j=1}^N}{\displaystyle \sum_{i=1}^{n_j}\frac{1}{2^{g_{ij}}}} $$

In this equation, **n**_**j**_ is the number of known ancestors for animal **j** and **g**_**ij**_ is the number of generations between animal **i** (ancestor) and animal **j**. The depth of the pedigree in each reference population was examined by computing G_e_ which is the expected number of generations from the base population, to the reference population if generation proceeded discretely [16].

The effective number of non-founders explains the amount of genetic diversity reduced by random genetic drift accumulated in non-founders’ generations and is calculated using the following equation [15]:

$$ {N}_{enf}={\left[\frac{1}{f_g}-\frac{1}{f_e}\right]}^{-1} $$

Where **N**_**enf**_ is the effective number of non-founders. Also, **f**_**g**_ and **f**_**e**_ are founder genome equivalents and founder equivalents, respectively.

The Inbupgf 90 program [17] was used for calculating regular inbreeding coefficients for individuals in the pedigree. Falconer and Mackay [18] established that the average inbreeding coefficient at a given generation t could be estimated using the following equation:

$$ {F}_t=1-{\left(1-\varDelta F\right)}^t $$

Where **ΔF** is the change in inbreeding from one generation to the next one or new inbreeding. González-Recio et al. [19] proposed to operate the above equation, to set the inbreeding coefficient for each individual, as represented below:

$$ \varDelta {F}_i=1-\sqrt[t]{1-{F}_i} $$

Where **F**_**i**_ is the individual coefficient of inbreeding and **t** is the equivalent complete generations [20].

The estimate of the effective number (N_e_) [17] can be calculated from *ΔF*, which can be easily computed by averaging the ΔF_i_ of *n* individuals included in a given reference subpopulation; therefore, effective number is obtained as:

$$ {N}_e=\frac{1}{2\varDelta F} $$

This way of computing effective population number is not dependent on the whole reference population mating policy, but on the matings carried out throughout the pedigree of each individual [21].

Generation interval (L) was calculated as the average age of the parents at the birth of their lambs. All the animals were grouped into three classes according to the inbreeding coefficients obtained by their pedigrees: the first class included non-inbred animals (*F=* 0); and the second and third classes included inbred animals (0< *F<* 0.05 and *F* ≥ 0.05, respectively). Moreover, the birth type (single, twin) and lamb sex (male, female) was considered for each of the lambs. Due to the low frequency of triple births, triple lambs were not included in this study.

Trend of inbreeding over time was estimated using the linear regression of individual inbreeding on the birth year using the Reg procedure of SAS [13]. The GLM procedure of SAS was used for determining the fixed factors which had significant effect on the traits investigated. After data verification, defective and doubtful records were deleted (e.g., lambs without weight records or with incomplete records of parentage or with registration numbers lower than the numbers of their parents were left out). The least-squares means were estimated for each trait using the Average Information Restricted Maximum Likelihood (AIREML) algorithm of the Wombat program [20] by fitting six single trait animal models which ignore or include additive direct and maternal genetic and permanent environmental effects. The statistical models included herd-year-season of lambing, lamb sex in 2 classes (male and female), age of dam at lambing in 6 classes (2–7 years old), birth type in 2 classes (single, twin), inbreeding in 3 groups (*F=* 0, 0< *F<* 0.05, *F* ≥ 0.05) and interaction between them. The most appropriate model for BW, 9 MW and YW included direct additive genetic and maternal permanent environmental effects and for WW included direct additive genetic effects as well as maternal additive genetic effects and for 6 MW included maternal and direct additive genetic effects as well as covariance between direct additive and maternal additive genetic effects.

## Results

### Analysis of pedigree

The analysis of pedigree revealed that inbreeding coefficient ranged from 0 to 27% with an average of 1.69%. Table 3 shows the summary statistics for body weight traits in different inbreeding classes of animals. The analysis of pedigree revealed that 60 animals out of 26990 (0.22%) had a high inbreeding coefficient (*F* ≥ 0.05) with a mean value of 22.10% while 24199 out of 26990 animals (89.66%) had medium inbreeding coefficient (0< *F<* 0.05). The remaining lambs (10.12%) were non-inbred. There were significant differences between three classes of inbreeding on BW and animals within first class of inbreeding had greater mean of the trait than two other groups (*P<* 0.05). The WW of animals within first class of inbreeding was higher than those of the lambs belonging to the second and third classes, but only differences were significant between first and third classes (*P<* 0.05). On the other hand, the 6 MW of animals within first class of inbreeding was significantly (*P<* 0.05) lower than those of the lambs in the second and third classes. Also, there were significant differences between three classes of inbreeding on 9 MW and animals within third class of inbreeding had greater mean of the trait than two other groups (*P<* 0.05). In addition, there were no significant differences between three classes of inbreeding on YW.

**Table 3:** **Distribution of records for body weight traits in different inbreeding classes of animals born between 1994 and 2011**

**Inbreeding class**		**Traits (kg)**								
	**BW**		**WW**		**6 MW**		**9 MW**		**YW**	
	**N**	**Mean ± SE**	**N**	**Mean ± SE**	**N**	**Mean ± SE**	**N**	**Mean ± SE**	**N**	**Mean ± SE**
F= 0	1536	3.88 ± 0.01^a^	802	22.46 ± 0.06^a^	348	34.62 ± 0.06^b^	33	39.06 ± 0.93^c^	12	50.20 ± 0.01^a^
0< F<0.05	8700	3.65 ± 0.01^b^	5903	22.12 ± 0.06^ab^	4503	36.23 ± 0.06^ab^	3095	45.51 ± 0.11^b^	1998	52.68 ± 0.15^a^
F ≥ 0.05	19	3.32 ± 0.23^c^	10	21.01 ± 0.06^b^	11	37.48 ± 0.06^a^	11	47.66 ± 0.88^a^	9	55.35 ± 1.32^a^

^**a,b,c**^Means with similar letters in each sub class within a column do not differ significantly at *P<0.05. BW*: birth weight, *WW*: 3-month weight, *6 MW*: 6-month weight, *9 MW*: 9-month weight, *YW*: yearling weight. *F*: inbreeding coefficient. *SE*: standard error.

**Figure 1:**
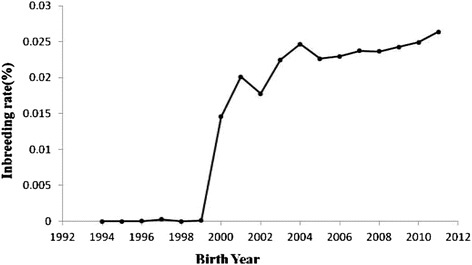
**Inbreeding trend over the years.**

**Figure 2:**
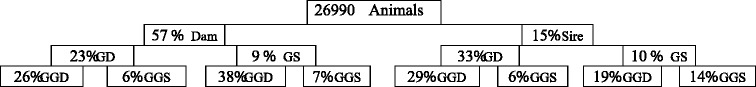
**Pedigree completeness up to 3 generations back.** *GD*: grand dam, *GS*: grand sire, *GGD*: great grand dam, *GGS*: great grand sire.

Table 4 shows the results of the pedigree analysis for the reference population in year groups. The f_e_ values were 4244, 3116 and 2965 during 1994–1999, 2000–2005 and 2006–2011, respectively. The f_g_ values were 4211, 2328 and 2118 during 1994–1999, 2000–2005 and 2006–2011, respectively. N_enf_ values were 10057, 9205 and 7422 during 1994–1999, 2000–2005 and 2006–2011, respectively. Therefore, this parameter was decreased over the years. The G_e_ values were 0.3571, 0.4545 and 0.5359 during 1994–1999, 2000–2005 and 2006–2011, respectively. MaxG_e_ were 1.625, 1.9375 and 2.77734 during 1994–1999, 2000–2005 and 2006–2011, respectively. The generation interval (L) was 2.15 years in Mehraban sheep. The average coancestries (AC) were 0.000118742, 0.000214769 and 0.000238527 during 1994–1999, 2000–2005 and 2006–2011, respectively. The effective population sizes (N_e_) were 363, 5080 and 5740 during 1994–1999, 2000–2005 and 2006–2011, respectively. Changes in inbreeding (ΔF) were 0.00137741, 0.00009843 and 0.00008711 during 1994–1999, 2000–2005 and 2006–2011, respectively. Figure 1 shows the trend of inbreeding coefficients over the years. The inbreeding trend was significantly positive over the years (*P<* 0.01) and its estimate was 0.002 ± 0.00003. Figure 2 shows the pedigree completeness up to 3 generations back. The first ancestor generation of all animals, included in the total data set used, was 15% sire and 57% dam complete.

**Table 4:** **The results of the pedigree analysis for the reference population of Mehraban sheep in year groups**

**Item/ year**	**1994-1999**	**2000-2005**	**2006-2011**	**Total years**
Number of animals	7745	9340	9905	26990
Founder equivalent (f_e_)	7244	3116	2965	-
Founder genome equivalent (f_g_)	4211	2328	2118	-
Effective number of non-founders (N_enf_)	10057	9205	7422	-
Effective population size (N_e_)	363	5080	5740	11963
Average number of discrete generation equivalents (G_e_)	0.3571	0.4545	0.5359	0.4188
Maximum number of discrete generation equivalents (MaxG_e_)	1.625	1.9375	2.77734	2.777734
Minimum number of discrete generation equivalents (MinG_e_)	0	0	0	0
Generation interval (L), years	2	2.18	2.27	2.15
Average coancestry (AC)	0.000118742	0.000214769	0.000238527	-
Changes in inbreeding (ΔF)	0.00137741	0.00009843	0.00008711	0.00004179

### Inbreeding effects

Table 5 shows the effects of inbreeding on body weight traits of the lambs according to the birth type. Single-born lambs showed no significant difference in all body weights except for 9-month weight which inbred animals were significantly different from non-inbreds (*P<* 0.05), while twin-born lambs showed a significant difference in all body weights. The BW of twin-born lambs was significantly different in three classes of inbreeding (*P<* 0.05) and animals within first class of inbreeding had greater mean of the trait than two other classes. Twin-born lambs in the first and second classes of inbreeding had greater WW than those of twin-born lambs in the third class (*P<* 0.05). The 6 MW of twin-born lambs within first class of inbreeding was significantly (*P<* 0.05) lower than those of lambs in the second and third classes (*P<* 0.05). Twin-born lambs in the third class of inbreeding showed significant differences for 9 MW and YW with lambs in the second class (*P<* 0.05).

**Table 5:** **Distribution of records for body weight traits in different inbreeding classes of animals grouped by the type of birth and born between 1994 and 2011**

**Birth type**	**Inbreeding class**		**Traits (kg)**								
		**BW**		**WW**		**6 MW**		**9 MW**		**YW**	
		**N**	**Mean ± SE**	**N**	**Mean ± SE**	**N**	**Mean ± SE**	**N**	**Mean ± SE**	**N**	**Mean ± SE**
	F= 0	1344	3.96 ± 0.47^a^	681	22.44 ± 4.92^a^	281	34.66 ± 8.13^a^	33	39.07 ± 8.29^b^	12	50.20 ± 0.01^a^
Single	0< F<0.05	6820	3.87 ± 0.68^a^	4476	21.98 ± 4.59^a^	3317	36.73 ± 7.47^a^	2198	45.78 ± 6.66^a^	1474	52.35 ± 8.73^a^
	F ≥ 0.05	11	3.85 ± 0.65^a^	6	22.37 ± 3.36^a^	6	36.22 ± 5.51^a^	6	46.57 ± 3.36^a^	4	52.60 ± 3.99^a^
	F= 0	192	3.33 ± 0.46^a^	121	22.61 ± 4.19^a^	67	34.41 ± 5.83b	-	-	-	-
Twin	0< F<0.05	1880	2.96 ± 0.77^b^	1427	22.55 ± 3.92^a^	1186	36.71 ± 6.24^ab^	897	44.76 ± 5.77^b^	524	53.65 ± 5.28^b^
	F ≥ 0.05	8	2.59 ± 0.83^c^	4	18.98 ± 1.69^b^	5	39.00 ± 2.53^a^	5	48.98 ± 0.67^a^	5	57.56 ± 1.28^a^

^**a,b,c**^Means with similar letters in each sub class within a column do not differ significantly at *P<*0.05. *BW*: birth weight, *WW*: weaning weight, *6 MW*: 6-month weight, *9 MW*: 9-month weight, *YW*: yearling weight. *F*: inbreeding coefficient. *SE*: standard error.

Table 6 shows the effects of inbreeding on body weight traits of the lambs according to the sex of lambs. Male lambs showed no significant differences in their WW, 6 MW and YW irrespective of the inbreeding coefficient. The BW of male lambs within third class of inbreeding was significantly lower than those of male lambs in the first and second classes (3.43 ± 1.05 kg vs. 3.77 ± 0.82 kg and 3.96 ± 0.51 kg, respectively). Also, 9 MW of male lambs within first class of inbreeding was significantly lower than those of male lambs in the second and third classes (40.64 ± 9.72 kg vs. 46.05 ± 6.43 kg and 46.80 ± 3.14 kg, respectively).

**Table 6:** **Distribution of records for body weight traits in different inbreeding classes of animals grouped by the sex of lamb and born between 1994 and 2011**

**lamb sex**	**Inbreeding class**		**Traits (kg)**								
		**BW**		**WW**		**6 MW**		**9 MW**		**YW**	
		**N**	**Mean ± SE**	**N**	**Mean ± SE**	**N**	**Mean ± SE**	**N**	**Mean ± SE**	**N**	**Mean ± SE**
	F= 0	730	3.96 ± 0.51^a^	373	22.48 ± 4.69^a^	152	35.30 ± 8.37^a^	19	40.64 ± 9.72^b^	12	50.20 ± 0.01^a^
Male	0< F<0.05	4264	3.77 ± 0.82^a^	2996	22.28 ± 4.16^a^	2265	36.81 ± 6.12^a^	1556	46.05 ± 6.43^a^	1042	53.16 ± 6.48^a^
	F ≥ 0.05	12	3.43 ± 1.05^b^	6	22.77 ± 2.30^a^	7	36.90 ± 5.67^a^	7	46.80 ± 3.14^a^	6	53.77 ± 3.66^a^
	F= 0	806	3.81 ± 0.46^a^	429	22.45 ± 4.63^a^	196	34.09 ± 7.26^b^	14	36.80 ± 0.83^c^	-	-
Female	0< F<0.05	4438	3.54 ± 0.76^a^	2917	21.95 ± 4.26^a^	2238	35.66 ± 5.69^b^	1539	44.97 ± 6.32^b^	956	52.17 ± 6.99^b^
	F ≥ 0.05	7	3.13 ± 0.76^b^	4	18.38 ± 2.68^b^	4	38.50 ± 1.00^a^	4	49.98 ± 0.82^a^	3	58.53 ± 0.47^a^

^**a,b,c**^Means with similar letters in each sub class within a column do not differ significantly at *P<*0.05. *BW*: birth weight, *WW*: 3-month weight, *6 MW*: 6-month weight, *9 MW*: 9-month weight, *YW*: yearling weight. *F*: inbreeding coefficient. *SE*: standard error.

Female lambs showed significant differences in their body weights based on their inbreeding coefficient. The BW and WW of female lambs within third class of inbreeding were significantly (*P<* 0.05) lower than those of lambs in the first and second classes. The 6 MW of female lambs showed a significant difference (*P<* 0.05) between third class of inbreeding and first and second classes (38.50 ± 1.00 kg vs. 34.09 ± 7.26 kg and 35.66 ± 5.69 kg; respectively). The 9 MW of female lambs showed a significant difference between all three classes of inbreeding (*P<* 0.05). On the other hand, the YW of female lambs showed significant difference between second and third classes of inbreeding (52.17 ± 6.99 kg vs. 58.53 ± 0.47 kg; *P<* 0.05).

### Regression coefficients of body weights

Table 7 shows the regression coefficients of body weights on inbreeding of lambs for a change of 1% in inbreeding. The regression coefficients of BW, WW, 6 MW and YW on lamb inbreeding were estimated to be −6.34 ± 0.69, −14.68 ± 5.33, 48.00 ± 9.43 and 98.65 ± 15.65, respectively (*P<* 0.01). Therefore, BW and WW decreased, respectively, by 6.34 g and 14.68 g due to 1% increase in inbreeding and 6 MW and YW increased, respectively, by 48.00 g and 98.65 g due to 1% increase in inbreeding (*P<* 0.01). The regression coefficient of 9 MW on lamb inbreeding was not significant.

**Table 7:** **Regression coefficients (±SE) of body weight traits (in grams) on inbreeding of lambs for a change of 1% in inbreeding**

**Item**	**BW**	**WW**	**6 MW**	**9 MW**	**YW**
Single	−1.84 ± 0.67**	−11.45 ± 6.45**	54.50 ± 12.35**	−22.00 ± 19.58	232.65 ± 27.96**
Twin	−7.22 ± 1.33**	−28.63 ± 9.16**	30.26 ± 13.68*	17.81 ± 16.52	19.59 ± 15.30
Male	−5.74 ± 0.99**	−9.23 ± 7.24	32.18 ± 12.87*	−10.72 ± 16.11	68.47 ± 17.83**
Female	−7.23 ± 0.94**	−21.66 ± 7.85**	65.23 ± 13.81**	7.23 ± 22.62	171.28 ± 29.97**
All	−6.34 ± 0.69**	−14.68 ± 5.33**	48.00 ± 9.43**	−4.09 ± 13.12	98.65 ± 15.65**

*BW*: birth weight, *WW*: 3-month weight, *6 MW*: 6-month weight, *9 MW*: 9-month weight, *YW*: yearling weight.*P<0.05. **P<0.01.

## Discussion

Reported estimates of lamb inbreeding effects on growth performance traits showed the same trend by other authors. Similar to the current results, some reported a lower regression coefficient for BW due to increase in inbreeding, e.g. Ghavi Hossein-Zadeh [22] observed a reduction of 0.009 kg for 1% increase of inbreeding in Iranian Moghani sheep; Selvaggi et al. [23] found a mean value of 0.019 kg in Leccese sheep; MacKinnon et al. [10], Analla et al. [24], Van Wyk et al. [9], Ercanbrack and Knight [25], Khan et al*.* [26] and Mirza et al. [8] reported regression coefficients of −0.027, −0.013, −0.008, −0.010, −0.008 and −0.007 kg, respectively. Reasons of variation in inbreeding effects could be due to differences between the breeds in allele separation, amount of genetic variation in the base population, management, and diversity of the founders of the flocks examined [10].

Similar to the current results, Van Wyk et al. [16] and Selvaggi et al. [23] reported significant reduction in WW of lambs due to 1% increase in inbreeding in different breeds of sheep and inconsistent with the current result, Ghavi Hossein-Zadeh [22] and Lamberson and Thomas [11] reported no significant reduction in WW due to inbreeding. Sex of lambs was a significant effect in the current analysis of inbreeding; but Barczak et al. [7] and Ghavi Hossein-Zadeh [22] observed non-significant differences between males and females. Barczak et al. [7] reported positive inbreeding effects on fourth week weight in a multi-breed sheep population and Ghavi Hossein-Zadeh [22] reported positive inbreeding effects on 6 MW and YW in Moghani sheep population. There are several methodological and biological factors which determine the estimated inbreeding impact on the performance traits. It is well known that both negative and positive effects exist. Therefore, in a population, bad and good inbreeding effects are mixed [4].

The results of this study indicated a significant increase in 6WW and YW of lambs due to 1% increase in inbreeding, but Ghavi Hossein-Zadeh [22] reported a significant reduction in YW of male lambs (0.357 kg). The possible explanation for the strong inbreeding depression observed for 6 MW and YW in this study was the higher heritability of this trait compared to other weight traits in Mehraban sheep [27].

The inbreeding level estimates are strongly determined by the two main factors: depth and completeness of pedigree and selection intensity. Selection intensity is often increased by the reproductive technologies being focused on a few superior animals (especially sires) and the application of advanced methods of genetic evaluation. Embryo transfer and artificial insemination technology currently allow the intensive use of the same sires, leading to increase in the relationship coefficient between animals, which help to the increase in inbreeding in this population. A high inbreeding level is observed for populations rebuilt from small number of founders [7], but on the other hand in this case the accuracy is strongly determined by the incompleteness of pedigrees [7]. Animal breeding emphasis on the genetic breeding values of the traits, used as criteria of sires and dams selection, can also raise the inbreeding coefficient, since relationship between animals tend to present similar genetic values, having as a consequence the selection of the most frequent relatives [3]. Average inbreeding estimates reported in this study were lower than reported estimates of Ghavi Hossein-Zadeh [22] in Moghani sheep (2.93%), Dorostkar et al. [28] in Moghani sheep (2.069%), Pedrosa et al. [3] in Santa Inês sheep in Brazil (2.33%). Van Wyk et al. [9] and Selvaggi et al. [23] reported high rates of inbreeding in Dormer sheep (16%) and Leccese sheep (8.1%), respectively. On the other hand, Eteqadi et al. [29] reported lower inbreeding (0.15%) in Guilan sheep. The lower inbreeding coefficient in the current sheep population compared with other studies could be due to the lack of designed mating programs and absence of selection, especially before 1999. The rapid increase in the rate of inbreeding in 1999–2000 could be resulted from the reduction in the number of sires. Similar to the current results, Eteqadi et al. [29], Ghavi Hossein-Zadeh [22], Dorostkar et al. [28], Pedrosa et al. [3] and Barczak et al. [7] reported positive trend for inbreeding over the years.

The generation interval was 2.15 years in the current population, and MacKinnon [10] reported the generation intervals of 2.65 and 4.28 years for different crossbred sheep. Van Wyk et al. [9], Pedrosa et al. [3], Ghavi Hossein-Zadeh [22] and Eteqadi et al. [29] reported generation intervals of 3.27, 3.70, 3.34 and 2.385 years for Elsenburg Dormer sheep, Santa Inês sheep, Moghani sheep and Guilan sheep, respectively. Lower estimates of generation interval would cause larger responses [30].

The f_e_ and f_g_ values are important parameters which can be used for management and control of small populations. Also, these parameters can increase the accuracy of changes in some parameters such as effective population size and inbreeding rate [31]. In a population, abundance of some forms of founder animals may be more than the others, in creating the next generation. This makes these animals have greater contributions than others in the population gene pool. The f_e_ parameter was calculated for correcting this item. The f_e_ value was 2965 in 2011 which was proportional to the increased number of animals (9905) in this year. This indicated the unequal contribution of founder animals in creating offspring. The most important limitation of f_e_ is converging genetic contribution of founder animals after several generations which will lead to remain f_e_ in a constant value.

The f_g_ parameter is an indicator for showing the unequal participation of founder animals and accidental loss of genes during transmission from parents to offspring. For this reason, the value of f_g_ is always lower than the f_e_ value and decreases rapidly over the time. As expected, the value of f_g_ was reduced over the studied years.

The G_e_ parameter is a factor to indicate the depth and quality of the pedigree. The G_e_ value was increased over the studied years. Therefore, this indicated the increase in pedigree information and its evolution over the years. The AC values were increased over the years. Animals’ AC predicts average inbreeding of future generation in a population. For this reason, this parameter can be used to calculate the effective population size in the future. High relative population size, meaning low variation in a population due to the reduction of variance between individuals, will lead to a decrease in the response to selection.

The effective size of population is described by the number of animals that mate in an ideal population and produce the same inbreeding increment of the population under study [32]. Evolutionary biologists have suggested that an effective population size in the range of 500–5000 is mandatory to secure evolutionary potential of natural populations [33]. The reduction in effective population size, as a direct outcome of reduction in genetic diversity, associates with various unfavorable phenomena such as inbreeding depression in fitness-related traits and an increased change in response to selection [18]. One problem with the N_e_ value is that the value indicates the number of breeding animals needed to produce the average ΔF and does not quantify the cumulative decrease in allelic diversity or changes in breeding structure from year to year (10). Hence, the value obtained for N_e_ is not comparable to measures of f_e_ and f_g_ [10]. Most breeding programs may try to minimize accumulation of inbreeding and quantify the increase by calculating the change in inbreeding per generation (∆F) [34] in order to decrease the possible negative effect of inbreeding on productive traits.

## Conclusion

In conclusion, average inbreeding was 1.69% in Iranian Mehraban sheep and an increasing trend for inbreeding was observed over the years. Both positive and negative inbreeding effects were found in the current study. Different methods are proposed to maximize response to selection in an acceptable level of inbreeding such as balanced use of animals as parents of the next generation, limiting the size of families and creating sub-lines. Implementation of these methods and use of designed mating system can help to obtain the optimal response to the selection by least accumulation of inbreeding in Mehraban sheep flock in the future. Overall, avoidance from inbreeding is a main objective on the management of vulnerable species and breeds and this is especially true with respect to these new findings in Mehraban sheep.
